# A modelling framework for translating discrete choice experiment results into cost‐effectiveness estimates: an application to designing tailored and scalable HIV and contraceptive services for adolescents in Gauteng, South Africa

**DOI:** 10.1002/jia2.26124

**Published:** 2023-07-18

**Authors:** Caroline Govathson, Lawrence C. Long, Colin A. Russell, Aneesa Moolla, Sophie Pascoe, Brooke E. Nichols

**Affiliations:** ^1^ Health Economics and Epidemiology Research Office Faculty of Health Sciences University of the Witwatersrand Johannesburg South Africa; ^2^ Department of Global Health Boston University School of Public Health Boston Massachusetts 02118 USA; ^3^ Department of Medical Microbiology Amsterdam University Medical Center Amsterdam The Netherlands

**Keywords:** discrete choice experiment, health economics, modelling, HIV, contraceptive, adolescents

## Abstract

**Introduction:**

South African youth and adolescents face a high burden of (Sexually Transmitted Infections) STIs, HIV and unintended pregnancies, but uptake of services remains low. To address this, tailored and scalable interventions are urgently needed. We developed a framework to fill the gap and translate the impact of facility‐level attributes into a cost‐effectiveness analysis for increasing HIV/contraceptive service uptake in adolescents using a discrete choice experiment (DCE).

**Methods:**

We used a DCE (*n* = 805) conducted in Gauteng, South Africa, which found that staff attitude, confidentiality, Wi‐Fi, subsidized food, afternoon hours and youth‐only services were preferred attributes of health services. Based on this, we simulated the uptake of services adapted for these preferences. We divided preferences into modifiable attributes that could readily be adapted (e.g. Wi‐Fi), and challenging to modify (more nuanced attributes that are more challenging to cost and evaluate): staff attitude and estimated the incremental change in the uptake of services using adapted services. Costs for modifiable preferences were estimated using data from two clinics in South Africa (2019 US$). We determined the incremental cost‐effectiveness ratio (ICER) for additional adolescents using services of 15 intervention combinations, and report the results of interventions on the cost‐effectiveness frontier.

**Results:**

Greatest projected impact on uptake was from friendly and confidential services, both of which were considered challenging to modify (18.5% 95% CI: 13.0%−24.0%; 8.4% 95% CI: 3.0%−14.0%, respectively). Modifiable factors on their own resulted in only small increases in expected uptake. (Food: 2.3% 95% CI: 4.0%−9.00%; Wi‐Fi: 3.0% 95% CI: −4.0% to 10.0%; Youth‐only services: 0.3% 95% CI: −6.0% to 7.0%; Afternoon services: 0.8% 95% CI: −6.0% to 7.0%). The order of interventions on the cost‐effectiveness frontier are Wi‐Fi and youth‐only services (ICER US$7.01−US$9.78 per additional adolescent utilizing HIV and contraceptive services), Wi‐Fi, youth‐only services and food (ICER US$9.32−US$10.45), followed by Wi‐Fi, youth‐only services and extended afternoon hours (ICER US$14.46–US$43.63).

**Conclusions:**

Combining DCE results and costing analyses within a modelling framework provides an innovative way to inform decisions on effective resource utilization. Modifiable preferences, such as Wi‐Fi provision, youth‐only services and subsidized food, have the potential to cost‐effectively increase the proportion of adolescents accessing HIV and contraceptive services.

## 1 INTRODUCTION

Existing models of care have failed to adequately address the gap in sexual health needs, access to―and uptake of―HIV and contraceptive services among adolescents in South Africa [1]. The rate of unintended pregnancy among adolescents remains high, with about a quarter of adolescent girls and young women giving birth before the age 20 [2, 3]. According to UNAIDS, the incidence rate for HIV infection among adolescents aged 10–19 was 54 per 1000 population in 2018 in South Africa with adolescent girls disproportionally affected [4]. Despite the high prevalence of HIV and pregnancy among adolescents, uptake of HIV and contraceptive services in this age group remains a challenge. The 2016 South African demographic health survey revealed that 31% of girls aged 15–19 years and 28% of AGYW aged 20–24 years had unmet contraceptive needs [5]. This underscores a critical need to increase adolescent access to both HIV and contraceptive services.

Many solutions have been offered to increase the uptake of HIV and contraceptive services by adolescents, including “youth‐friendly clinics” and school health programmes with limited success [1, 6, 7]. Children 12 years and older have the right to reproductive health, including access to contraceptives and HIV/AIDS testing without the consent of a parent or guardian [8]. There, however, remain many barriers to adolescents’ access and uptake of reproductive health services [9, 10, 11]. South Africa urgently needs tailored, scalable interventions to address both HIV infection and early pregnancy prevention for young people, in particular for young women. Decisions on how to tailor and efficiently scale interventions with regard to cost and uptake are often difficult to make due to limited data on adolescent preferences, impact and cost of different interventions.

Discrete choice experiments (DCEs) are a study design increasingly used in health economics to explore the relative importance of different attributes to decisions to access and utilize certain goods, services or programmes [12, 13]. They can help understand whether particular attributes can predict the uptake of services and the relative importance of certain attributes to this uptake [14]. The results can help policymakers understand the impact of making certain changes to current services on service acceptability or uptake. Research has been conducted to determine the preferences of school‐going youth for HIV and contraceptive services through the use of a DCE [15]. Key service characteristics, including staff attitude, confidentiality and value‐added services like availability of Wi‐Fi, food and youth‐only waiting areas, were found to be key attributes affecting the decision to access care. The cost and expected effectiveness of altering these key attributes to improve the likelihood that the youth access health services is, however, unknown.

When designing interventions using preference data from a DCE, information on incremental uptake and how much it costs to implement these interventions is essential [16, 17]. This information allows us to compare the different interventions and select only the most cost‐effective, scalable interventions for trialling. Traditionally, trial data, stakeholder opinions, and observational data of comparable scenarios are used with some underlying assumptions to predict uptake and cost [16, 17, 18, 19]. We describe an approach that can be used to close the gap between the DCE methodology and the implementation or trialling of different targeted programmes.

In order to guide future trials or implementation, we used the results of the DCE with school‐going adolescents [15] to model the expected costs and impact (in terms of an increase in the proportion of adolescents accessing health services) of attributes of health services. We used coefficients of expected uptake of HIV and contraceptive services from a recently conducted DCE [20], as an estimated outcome measure as part of a cost‐effectiveness analysis. This information can be used to determine which interventions or combination of interventions should take priority within limited budgets.

## 2 METHODS

This work builds on a DCE that was conducted to estimate the preferences of school‐going adolescents for accessing HIV and contraceptive services [15]. HIV and contraceptive services were defined according to the integrated school health policy as: HIV counselling and testing (HCT), linkage to care and treatment, sexual and reproductive health services, including the provision of dual protection (to prevent pregnancy and STIs, including HIV infection). The DCE was conducted in 10 high schools situated in neighbourhoods of varying socio‐economic status in Gauteng South Africa between July 2018 and September 2019. A total of 805 students completed the survey for the adolescent DCE study. Over two‐thirds (68%) were female, and two‐thirds (66%) were aged 15–17 years. Details of the DCE will be published elsewhere [15].

**Figure 1:**
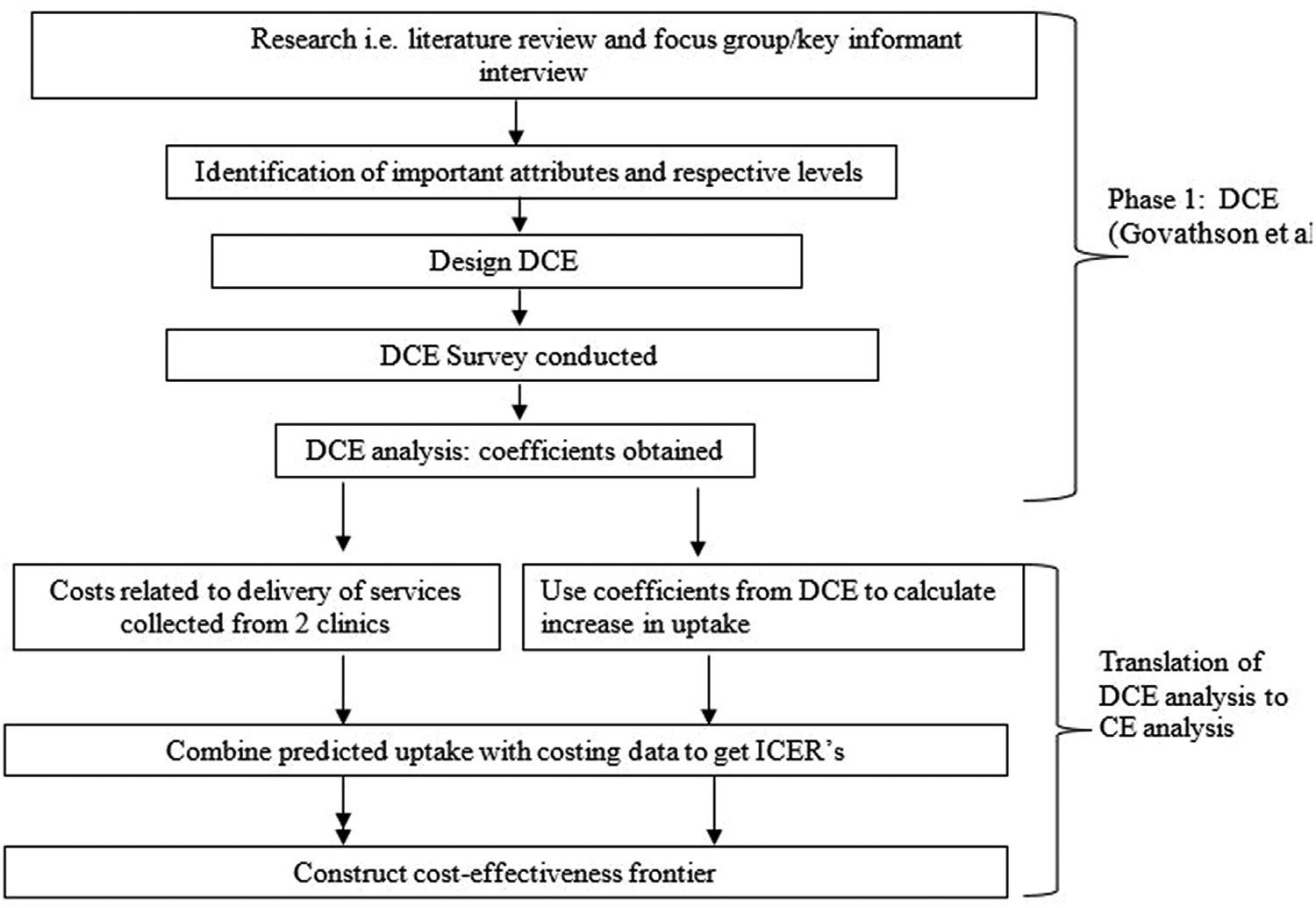
Translation of discrete choice experiment results to cost‐effectiveness results.

In the primary DCE, eight attributes were evaluated, each with two to four levels. These attributes included location, operating hours, healthcare provider characteristics, staff attitude, confidentiality, value‐added services, types of services offered and cost. Participants were asked their choice from options of hypothetical services offered. A conditional logit model was used to determine the relationship between each level of an attribute and choice. Six factors were found to be facilitators for uptake [15]. These were the six attributes and levels that showed significant change in the probability of a choice being selected. Staff attitude and confidentiality, access to subsidized food, youth‐only waiting areas, afternoon services and the presence of free Wi‐Fi were the preferences that were significantly associated with choice in the DCE. We used these results to identify combinations of interventions that could be used to increase the uptake of HIV and contraceptive services by adolescents. The attributes were categorized into modifiable (easier to implement, and to cost and evaluate): subsidized food, Wi‐Fi, youth‐only services and afternoon services (after school hours); and challenging to modify (more nuanced attributes that are more challenging to cost and evaluate): friendliness and confidentiality. We looked at the potential increase in service uptake across the full set of mutually exclusive combinations of all six of these factors (Table 1). The complete cost‐effectiveness analysis was, however, only done for the four factors that could be reasonably costed in a generalizable way, modifiable factors. These four factors were placed into all possible combinations of between one and four attributes. This resulted in 15 different modifiable mutually exclusive intervention combinations that could be evaluated to see if they are cost‐effective options. See Figure 1 for full methods illustration.

**Table 1:** Uptake of HIV and contraceptive service projections for intervention scenarios.

	DCE coefficient	% usage increase in uptake from baseline	Standard error	*p*‐value	Confidence interval	Total % of adolescents using HIV services as proportion of all clinic users
Baseline						30.60
Afternoon	0.01	0.82	0.03	0.80	−5.62 to 7.27	31.42
Wi‐Fi	0.03	2.71	0.04	0.45	−4.25 to 9.67	33.31
Youth waiting area	0.00	0.29	0.03	0.93	−6.17 to 6.74	30.89
Food	0.02	2.25	0.03	0.49	−4.10 to 8.61	32.85
Friendly	0.19	18.54	0.03	0.00	13.44−23.63	49.14
Confidential	0.08	8.35	0.03	0.00	3.09−13.60	38.95
Wi‐Fi and food	0.11	10.70	0.05	0.03	10.00−20.00	41.30
Food and youth	0.08	8.30	0.04	0.06	0.00−16.00	38.90
Youth and Wi‐Fi	0.09	9.00	0.04	0.07	0.00−18.00	39.60
Afternoon and Wi‐Fi	0.09	9.00	0.04	0.02	1.20−17.00	39.60
Afternoon and youth‐only	0.07	6.90	0.04	0.08	−0.87 to 14.67	37.50
Food and afternoon	0.08	8.00	0.03	0.02	1.20−16.40	38.60
Youth‐only, afternoon and Wi‐Fi	0.17	16.84	0.06	0.01	4.59−29.08	47.44
Wi‐Fi, food and youth	0.17	16.70	0.06	0.01	4.50−28.80	47.30
Wi‐Fi, food and afternoon	0.17	17.20	0.05	0.00	6.90−27.48	47.80
Food, afternoon and youth	0.15	14.80	0.05	0.00	5.48−24.24	45.40
Wi‐Fi, food, afternoon and youth	0.23	23.00	3.65	0.00	10.60−35.00	53.60
Youth‐only, Wi‐Fi, afternoon and friendly	0.49	49.42	0.04	0.00	40.86−57.99	80.02
Youth‐only, Wi‐Fi, afternoon and friendly	0.38	37.99	0.05	0.00	28.60−47.38	68.59
Youth‐only, Wi‐Fi, food, afternoon, friendly and confidentiality	0.55	55.00	0.05	0.00	46.00−65.00	85.60

### 2.1 Translation of DCE results to uptake

We used coefficients from the DCE [15] to estimate the expected increase in the uptake of HIV and contraceptive services as the outcome measure for a cost‐effectiveness analysis. We defined the baseline to be the set of attributes that most closely reflect current practice (location: clinic, time: morning, staff attitude: unfriendly, confidentiality: none, incentives: none and provision of all health services). Assumptions about baseline service characteristics were made from literature and conversations with stakeholders [1, 21]. To estimate an increase in uptake of services, we calculated the change of probability of uptake of using services from baseline levels when changes are made to the next level of each attribute (i.e. the incremental change in uptake). We modelled attributes, which had shown a statistically significant effect on the probability of an attribute being chosen in the DCE.

The logit probability of choosing alternative *i* rather than alternative *j* is given by the equation:

$$\begin{array}{c} Pi=e\beta^{\prime }xi\\ \sum e\beta^{\prime }xj. \end{array}$$

The change in the probability of choosing to use services given the baseline levels say, clinic, and morning services is then (provided that all other attributes remain equal) given by the above equation [22]. We then estimated the number of additional adolescents who would be expected to utilize HIV and contraceptive services per month over a 1‐year period.

### 2.2 Costs

Economic costs related to the delivery of a modified package of clinic interventions delivered with the aim of increasing the utilization of HIV and contraceptive services were estimated from a health system perspective. We assumed linear scale‐up in costs to understand what the relative cost‐effectiveness of these interventions would be in a typical public health clinic at scale.

Baseline costs were estimated from cost data collected from two clinics (one government primary health clinic [PHC] and one South African non‐profit organization providing comprehensive PHC services with Department of Health Support) through an external study focused on the costing of primary health services [23, 24]. Costs were imputed per month over a period of 12 months. The methods for the costing and the description of these PHCs are elsewhere [23, 24, 25]. For each of the intervention scenarios, only the additional costs associated with the modification were calculated. The additional costs associated with the modification were allocated to the additional adolescent patients expected to utilize the clinic following the changes.

The changes in cost in categories of assets, overheads, staff (clinical and non‐clinical) and supplies for each package of interventions were calculated based on a number of assumptions regarding implementation and current local costs of implementing those changes.

Utilities, such as electricity, water and sewage costs, were allocated based on the total number of monthly visits. Staff costs for the afternoon and for youth‐only services were calculated based on the number of patients seen and period of time the facility would be open in the afternoon. Food costs were calculated for all adolescents but the total cost was billed only to the additional adolescent patients so that the incremental cost included the cost of food to all adolescents. Extra space for youth‐only services or afternoon services was calculated using average‐size counselling rooms.

We calculated a cost per additional adolescent patient expected to utilize HIV and contraceptive services as a consequence of the clinic modifications and the total costs required at the facility level to implement the intervention. All costs were updated to 2019 South African Rand (ZAR) and converted to USD using the mid‐year exchange rate 1 USD = 14 ZAR [26]. Analysis of costs was done using Microsoft Excel. Table A1 summarizes sources of and adjustments to cost for each intervention scenario. Table A2 summarizes the average cost and cost per category by intervention scenario.

### 2.3 Cost‐effectiveness ratios

In order to generate incremental cost‐effectiveness ratios (ICERs), the predicted probability analysis from the DCE was combined with costing data for selected intervention scenarios. We conducted an incremental cost‐effectiveness analysis, comparing interventions ordered by total cost, and determined the set of non‐dominated strategies for increasing service uptake. We also determined the optimal order of implementation across the cost‐effectiveness frontier. Strategies that cost more and did not increase uptake of services were considered “dominated” (e.g. costlier and less or equally effective) and, therefore, removed from the comparison. When the intervention increased the uptake of the services and was also less costly, we called it the “dominant” intervention. For interventions that increased uptake and also the cost, an ICER was calculated to compare the interventions, or a cost per additional adolescent expected to access services. To determine the most cost‐effective strategies, we used the efficiency frontier approach. A cost‐effectiveness frontier representing the efficient combination of interventions of the 15 strategies assessed was constructed. Interventions below the frontier are not considered cost‐effective at any threshold [27].

In the incremental analysis, strategies were compared until ultimately, only the non‐dominated strategies were left and these were ordered according to their cost‐effectiveness ratio, lowest to highest, referencing which strategies should be adopted in incremental order dependent on the availability of resources [27, 28].

### 2.4 Sensitivity analysis

To account for possible over‐estimation of real‐world uptake from the DCE, we conducted a sensitivity analysis of the cost‐effectiveness of all interventions using the lower bound of the confidence intervals on the uptake calculation. This was done to identify how the relative cost‐effectiveness and cost‐effectiveness frontier would change with a different set of possible outcomes.

### 2.5 Ethics

As this was a modelling study based on results from published studies that did not involve human subjects, approval from an institutional review board was not necessary.

## 3 RESULTS

### 3.1 Preferences (results from the DCE)

Results from the DCE that informed this work showed that the most preferred service characteristics that facilitated service uptake by adolescents were friendly healthcare providers and private and confidential services. Students preferred accessing services in the afternoon (after school hours) compared to the morning. Youth‐only services, Wi‐Fi and access to food were likely to increase the probability of students utilizing services. Students preferred the more comprehensive package of services (family planning and contraceptive services) or all health services as opposed to services that only provided condoms or HCT [15].

### 3.2 Uptake projections

**Figure 2:**
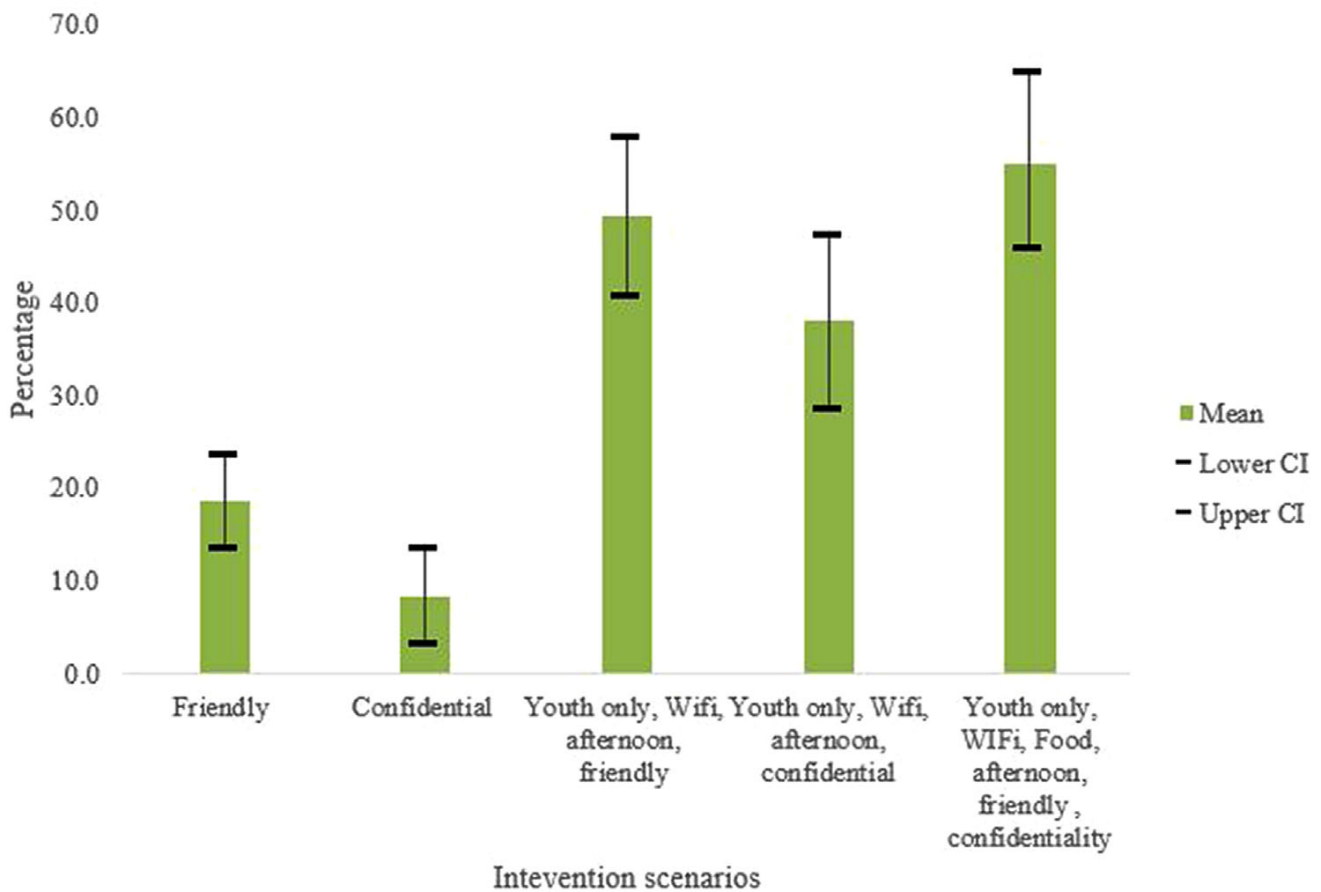
Uptake of HIV and contraceptive services projections for scenarios that include at least one challenging to modify attribute. Abbreviation: CI, confidence interval.

Results from the simulations conducted indicate that uptake of HIV and contraceptive services may increase as different service attributes are added to the baseline standard of care. The more challenging to modify factors, such as friendly healthcare providers and confidentiality of services, were projected to have the largest impact on the uptake, an increase of (18.5%; 95% CI 13.0%−24.0%; and 8.4%; 95% CI 3.0%−14.0%), respectively. Combining these challenging to modify preferences with three modifiable preferences (Wi‐Fi, food, youth‐only services and afternoon services) projected a statistically significant increase in uptake by 55.0% (95% CI 46.0%−65.0%) from baseline (Figure 2).

**Figure 3:**
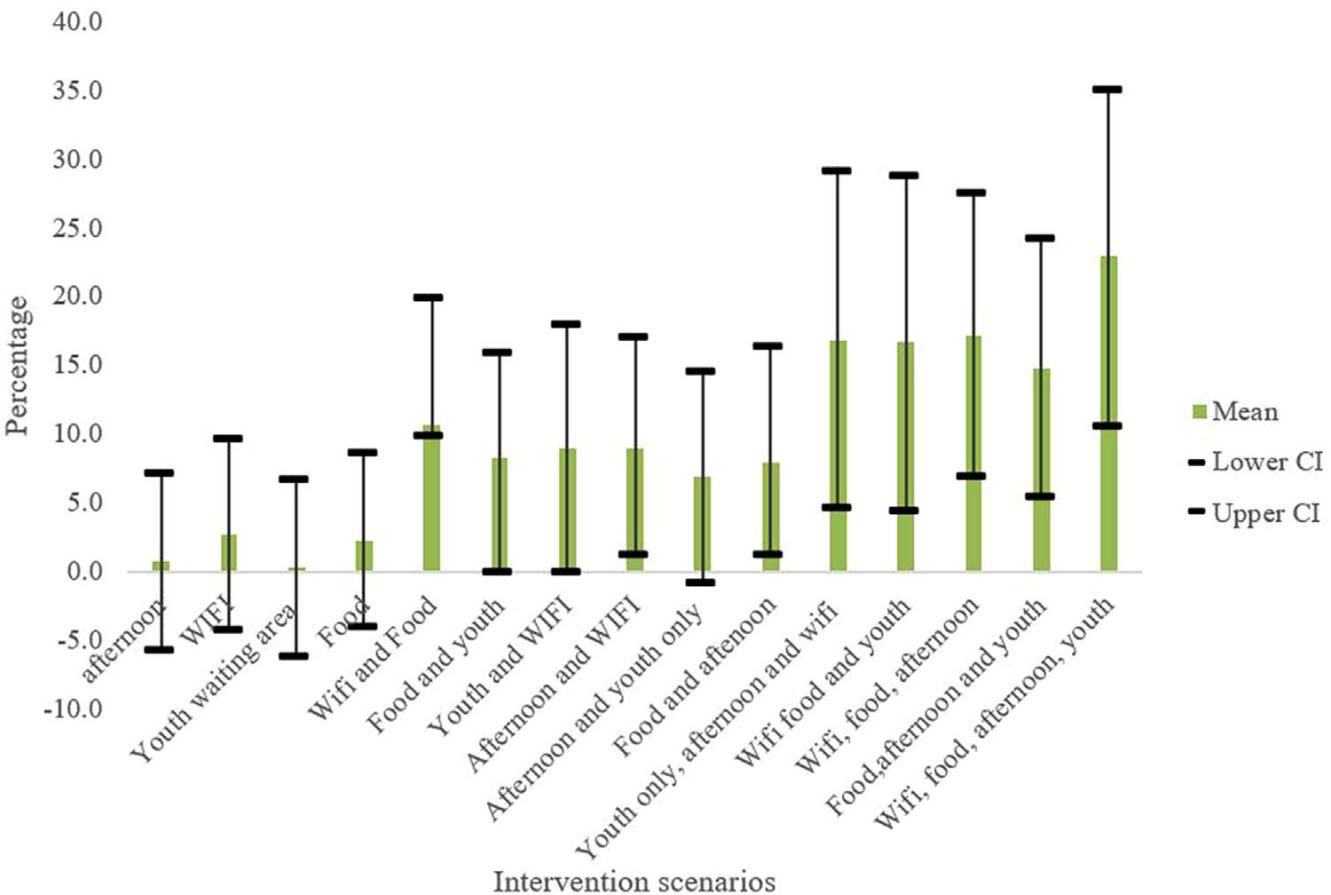
Uptake of HIV and contraceptive services projections for modifiable intervention scenarios. Abbreviation: CI, confidence interval.

Only modifiable attributes were included in the final cost‐effectiveness analysis. On their own, the provision of afternoon services, or youth‐friendly services or Wi‐Fi only or food only did not yield a statistically significant increase in rates of uptake of services. The least effective strategies were, in ascending order of efficacy, youth‐only services, afternoon services, access to subsidized food and provision of Wi‐Fi separately. The combination of all four attributes, however, (Wi‐Fi, food, afternoon services and youth‐only) provided the highest increase in uptake of 23.0% (95% CI 11.0%−35.0%) (Figure 3). This was followed by different combinations of just three attributes, 17.0% for any combination of the three attributes (Table 1). Combining only two preferred modifiable attributes might increase uptake by between 7.0% and 11.0% depending on the combination of preferred attributes (Table 1). The number of adolescents utilizing HIV and contraceptive services at baseline was higher at Clinic B than at Clinic A, therefore, as a fraction, Clinic A had more additional adolescents utilizing services.

### 3.3 Cost and cost‐effectiveness

The cost per additional adolescent utilizing services varied between the clinics depending on which interventions or package of interventions might be implemented. Clinic A costs ranged from $10.20 to $202.90 (Table 2) and Clinic B from $7.00 to $116.50 for Clinic B (Table 3). Table A1 summarizes sources of and adjustments to cost for each intervention scenario. Table A2 summarizes the average cost and cost per category by intervention scenario.

**Table 2:** Clinic A: Cost‐effectiveness of interventions to increase adolescent uptake of HIV and contraceptive services.

Number	Additional adolescents per month	Total number of adolescents expected to accessing services	% change from baseline	Cost per patient	Total cost	ICER/additional adolescent utilizing services
Wi‐Fi	41	1572	2.7	$12.56	$516.65	Dominated
Youth‐only	4	1535	0.3	$202.91	$811.63	Dominated
Afternoon	13	1544	0.8	$78.47	$1020.14	Dominated
Food	34	1565	2.3	$31.54	$1072.28	Dominated
Wi‐Fi and youth‐only	138	1669	9.0	$10.62	$1465.56	$9.78
Wi‐Fi and food	168	1699	10.7	$10.15	$1705.92	Weakly dominated^a^
Food and youth‐only	122	1653	8.3	$15.23	$1858.50	Dominated
Wi‐Fi and afternoon	142	1673	9.3	$18.33	$2603.24	Weakly dominated^a^
Wi‐Fi, food and youth‐only	260	1791	17.0	$10.54	$2739.87	$10.45
Afternoon and youth‐only	106	1637	6.9	$27.03	$2864.78	Dominated
Wi‐Fi, afternoon and youth‐only	230	1761	17.0	$13.68	$3147.26	Dominated
Food and afternoon	138	1669	9.0	$24.94	$3441.86	Dominated
Food, afternoon and youth‐only	230	1761	15.0	$18.09	$4160.93	Dominated
Wi‐Fi, food and afternoon	260	1791	17.0	$16.35	$4251.24	Dominated
Wi‐Fi, food, afternoon and youth‐only	352	1883	23.0	$19.20	$6757.95	$43.67

^a^A strategy is said to be weakly dominated when it is less effective and costlier than another strategy or is equally effective but costlier than another strategy.

**Table 3:** Clinic B: Cost‐effectiveness of interventions to increase adolescent uptake of HIV and contraceptive services.

	Additional adolescents per month	Total number of adolescents expected to accessing services	% change from baseline	Cost per additional adolescent	Total cost	ICER/per additional adolescents utilizing services
Wi‐Fi	49	1857	2.7	$10.66	$522.69	Weakly dominated^a^
Youth‐only	5	1813	0.3	$116.50	$611.08	Dominated
Wi‐Fi and youth‐only	163	1971	9.0	$7.01	$1141.87	$7.01
Afternoon	15	1823	0.8	$77.58	$1150.59	Dominated
Food	41	1849	2.3	$30.83	$1254.73	Dominated
Food and youth‐only	145	1953	8.0	$11.43	$1653.56	Dominated
Afternoon and youth‐only	125	1933	6.9	$13.79	$1720.92	Dominated
Wi‐Fi and food	199	2007	10.7	$8.97	$1785.57	Weakly dominated^a^
Wi‐Fi and afternoon	168	1976	9.3	$7.45	$1253.69	Dominated
Food and afternoon	163	1971	9.0	$14.83	$2413.33	Dominated
Wi‐Fi, food and youth‐only	307	2115	17.0	$8.10	$2490.46	$9.32
Wi‐Fi, afternoon and youth‐only	271	2079	15.0	$8.45	$2293.28	Dominated
Wi‐Fi, food and afternoon	307	2115	17.0	$12.73	$3914.33	Dominated
Food, afternoon and youth‐only	271	2079	15.0	$11.15	$3025.94	Dominated
Wi‐Fi, food, afternoon and youth‐only	416	2224	23.0	$9.76	$4059.62	$14.45

^a^A strategy is said to be weakly dominated when it is less effective and costlier than another strategy or is equally effective but costlier than another strategy.

**Table A1:** Summary of costs, assumptions and sources of cost data for each scenario

Cost categories	Methods	Assumptions	Source
Total PHC headcounts were calculated using monthly headcounts over a period of 12 months (2018)			
Staff (clinical and non‐clinical)	Top‐down approach for staff	Additional adolescents would bear the cost of all additional staff at the facility as a result of intervention.	Data collected for Long et al [24]
Consumables/supplies	Top‐down. Calculated using monthly expenditure on supplies. This was shared among all PHC patients. (Total spent on consumables/total number of patients).	Maintained the per patient cost of consumables at the clinic level for the new adolescent patients obtained.	Data collected for Long et al [24]
Overheads	Overhead costs were calculated though a top‐down approach. Additional overhead costs, that is extra space for youth‐only, were all shared by additional adolescents.	A separate space the size of a single counselling room at the facility was allocated for youth‐only services. Overheads were calculated by multiplying total overhead expenditure by proportion of new adolescents over total PHC count.	Data collected from Long et al [24]
Assets	Bottom‐up inventory of all assets in clinic and shared among all patients.	Shared the total cost of assets among all patients as no new assets would be necessary for intervention.	Data collected from Long et al [24]
Wi‐Fi		Wi‐Fi—Project Isizwe (free Wi‐Fi for Tshwane clinics average costs). Costs of supply to a clinic for 1 month shared by all adolescents.	Data collected from Long [24]^a^
Subsidized food		All adolescents presenting received subsidized food but additional adolescents bore the cost. A total of ZAR 10 was cost for each adolescent patient. The assumption was around the provision of subsidizing food at 10 Rand. This assumption discussed and validated in stakeholder meetings with the Department of Education.	Costs from et al [39]

^a^ Project ISIZWE providing public Wi‐Fi access in South Africa.

**Table A2:** Clinic B average cost and cost per category in USD by intervention to increase the utilization of HIV and students

	**Baseline fixed cost per month**	**Baseline fixed cost per visit**	**Afternoon**	**Wi‐Fi**	**Youth**	**Food**	**Wi‐Fi and youth**	**Afternoon and youth**	**Food and youth**	**Afternoon and food**	**Food and Wi‐Fi**	**Afternoon and Wi‐Fi**	**Afternoon, youth and food**	**Afternoon, Wi‐Fi and food**	**Food, Wi‐Fi and youth**	**Afternoon, youth and Wi‐Fi**	**Afternoon, youth, food and Wi‐Fi**
Assets	1286.36	0.21	_	_	_	_	_	_	_	_	_	_	_	_	_	_	_
Overheads	7990.39	1.33	16.89	_	116.42	_	3.82	4.78	3.78	1.12	_	0.13	2.30	0.06	2.05	2.30	1.54
Staff (total)	55,458.66	9.20	60.60	_	_	_	_	8.93	_	6.94	_	4.17	4.17	6.83	_	4.17	5.04
Clinical	40,228.78	6.67	_	_	_	_	_	_	_	_	_	_	_	_	_	_	
Non‐clinical(overhead)	15,229.88	2.53	_	_	_	_	_	_	_	_	_	_	_	_	_		
Supplies	464.36	0.08	0.08	0.08	0.08	0.08	0.08	0.08	0.08	0.08	0.08	0.08	0.08	0.08	0.08	0.08	0.08
Wi‐Fi	_	_	_	10.58	_	_	3.19	_	_	7.69	6.28	3.07	_	1.69	1.90	1.90	0.09
Food	_	_	_	_	_	30.75	_	_	7.94	_	2.61	_	4.60	4.07	4.07	_	3.01
Total	120,658.44	10.81	77.57	10.66	116.50	30.82	7.01	13.79	11.80	14.83	8.97	7.45	11.15	12.73	8.10	8.45	9.76

Only costs that directly affect the implementation of intervention to increase uptake were considered.

The addition of subsidized food had the third highest cost per additional adolescent utilizing HIV and contraceptive services at US$31.54 for Clinic A and US$30.83 for Clinic B. This was because we cost the food to benefit all adolescents utilizing services but the cost was borne only by the additional adolescents utilizing services. Provision of services in the afternoon outside school hours has the next highest cost per additional adolescent utilizing services (US$78.54 per additional adolescent at Clinic A and US$77.56 at Clinic B). Providing youth‐only services would be the costliest per additional adolescent accessing HIV and contraceptive services with costs for Clinic A at US$202.91 and Clinic B at US$116.50. Combining all modifiable attributes was US$19.20 for Clinic A and US$9.76 for Clinic B per additional adolescent utilizing services.

**Figure 4:**
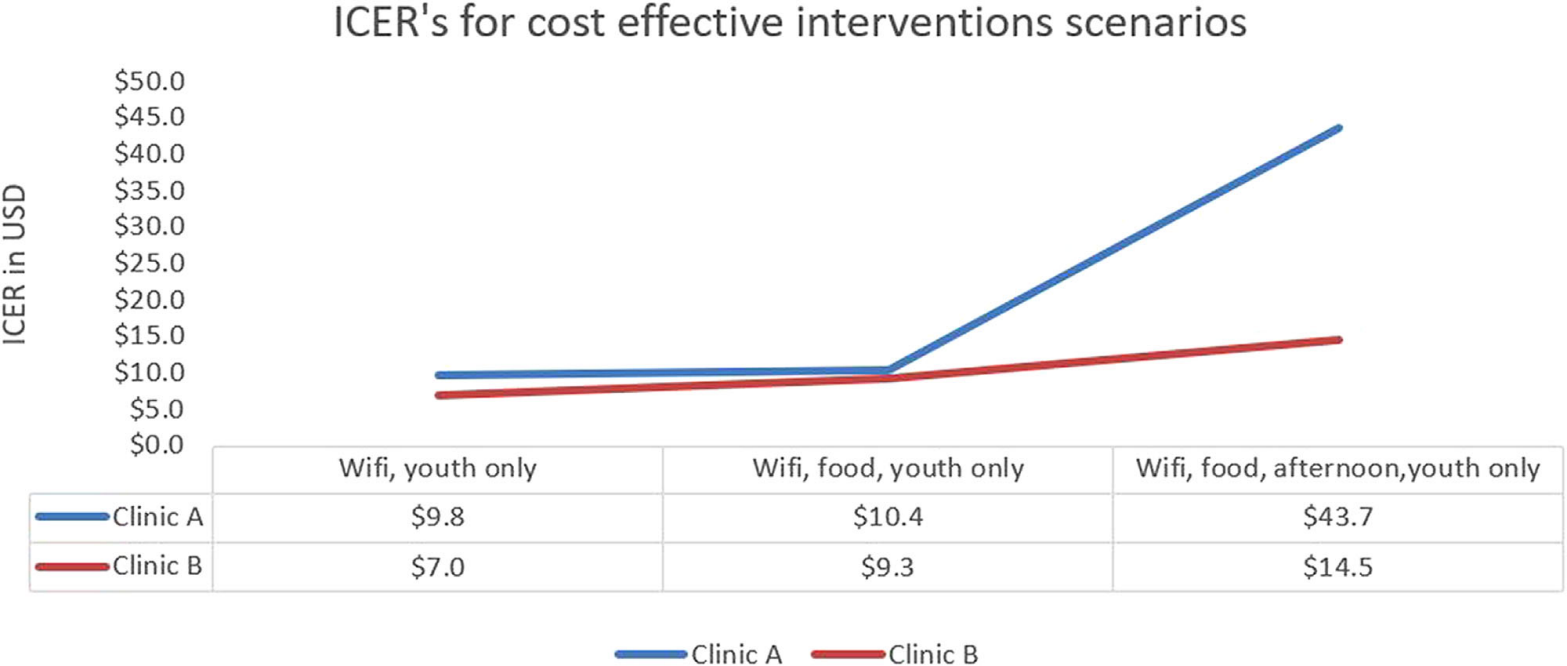
Incremental cost‐effectiveness ratios on the cost‐effectiveness frontier.

Figure 4 shows all non‐dominated intervention scenarios for the two clinics, ranked in order of decreasing cost‐effectiveness. For both clinics, the ICER for youth‐only services and Wi‐Fi was the first intervention on the cost‐effectiveness frontier. The incremental cost per additional adolescent using the services for Clinic A ranges from US$9.78 for (Wi‐Fi and youth‐only services) and US$43.67 for a combination of all four modifiable attributes. The same order is for Clinic B, though with lower incremental costs per additional adolescent using services―US$7.01 for (Wi‐Fi and youth‐only services) and US$14.50 for all four modifiable attributes combined.

### 3.4 Sensitivity analysis

In our sensitivity analyses, (Wi‐Fi and food) and (Wi‐Fi, food, afternoon and youth‐only) were the interventions on the cost‐effectiveness frontier. For both clinics, the ICER for (Wi‐Fi and food) was the first intervention on the cost‐effectiveness frontier, $1.10 and $6.23 for Clinic A and B, respectively. Followed by all four modifiable attributes at $566.79 and $212.69, Clinic A and B, respectively (Tables 1, 2, A3 and A4).

**Table A3:** Clinic A one‐way sensitivity analysis by lower confidence interval bound of uptake for cost‐effectiveness of interventions to increase adolescent uptake of HIV and contraceptive services

	Additional adolescents	New total adolescents	%	Cost per additional adolescent patient	Total cost	ICER/per additional adolescents utilizing services
Wi‐Fi	1	1532	−4.3	$535.81	$535.81	Dominated
Youth‐only	1	1532	−6.2	$929.29	$929.29	Dominated
Afternoon	1	1532	−5.6	$1015.51	$1015.51	Dominated
Food	1	1532	−4.1	$1117.98	$1117.98	Dominated
Food and youth‐only	1	1532	0.0	$1198.75	$1198.75	Dominated
Wi‐Fi and youth‐only	1	1532	0.0	$1497.53	$1497.53	Dominated
Wi‐Fi and afternoon	18	1549	1.2	$87.96	$1615.62	Dominated
Wi‐Fi and food	153	1684	10.0	$11.52	$1763.88	$1.10
Afternoon and youth‐only	18	1549	1.2	$109.35	$2008.40	Dominated
Wi‐Fi, food and youth‐only	69	1599	4.5	$30.88	$2126.94	Dominated
Food and afternoon	1	1532	−0.9	$3252.15	$3252.15	Dominated
Wi‐Fi, afternoon and youth‐only	70	1601	4.6	$50.73	$3560.49	Dominated
Food, afternoon and youth‐only	84	1614	5.5	$50.88	$4267.50	Dominated
Wi‐Fi, food and afternoon	106	1636	6.9	$41.29	$4361.22	Dominated
Wi‐Fi, food, afternoon and youth‐only	162	1693	10.6	$42.95	$6969.09	$566.79

A strategy is said to be weakly dominated/dominated when it is less effective and costlier than another strategy or is equally effective but costlier than another strategy.

**Table A4:** Clinic B one‐way sensitivity analysis by lower confidence interval bound of uptake for cost‐effectiveness of interventions to increase adolescent uptake of HIV and contraceptive services

	Additional adolescents	Total adolescents	%	Cost per additional adolescent patient	Total cost	ICER/per additional adolescent utilizing services
Wi‐Fi	1	1809	−4.3	$11.01	$11.01	Dominated
Food	1	1809	−4.1	$31.82	$31.82	Dominated
Afternoon	1	1809	−5.6	$80.07	$80.07	Dominated
Youth‐only	1	1809	−6.2	$630.40	$630.40	Dominated
Wi‐Fi and afternoon	22	1830	1.2	$1166.11	$856.85	Dominated
Wi‐Fi and youth‐only	1	1809	0.0	$81.54	$1166.11	Dominated
Afternoon and youth‐only	22	1830	1.2	$10.17	$1769.81	Dominated
Wi‐Fi and food	181	1989	10.0	$1907.59	$1840.26	$6.18
Food and youth‐only	1	1809	0.0	$27.82	$1907.59	Dominated
Wi‐Fi, food and youth‐only	81	1889	4.5	$28.34	$2264.65	Dominated
Wi‐Fi, afternoon and youth‐only	83	1891	4.6	$2478.44	$2350.82	Dominated
Food and afternoon	1	1809	−0.9	$34.94	$2478.44	Dominated
Food, afternoon and youth‐only	99	1907	5.5	$32.21	$3463.00	Dominated
Wi‐Fi, food and afternoon	125	1933	6.9	$21.64	$4019.66	Dominated
Wi‐Fi, food, afternoon and youth‐only	192	2000	10.6	$571.64	$4148.30	$212.73

A strategy is said to be weakly dominated/dominated when it is less effective and costlier than another strategy or is equally effective but costlier than another strategy.

## 4 DISCUSSION

We have described a methodology to estimate the cost‐effectiveness of potential service delivery mechanisms for HIV and contraceptive services for adolescents, utilizing results of a DCE. Typically, cost‐effectiveness analyses are conducted after costly and time‐consuming trials or large‐scale observational studies have been completed. However, our pre‐intervention assessment of potential cost‐effectiveness based on the stated preferences of the target audience and an estimate of costs for each intervention can guide more efficient trial planning by including only the arms that are expected to be on the cost‐effectiveness frontier in trial designs. Our results can also guide programme planning and scale‐up efforts.

Our study's strength and innovation lie in using data from the DCE to calculate potential changes in service uptake from the current standard of care if different interventions were implemented, along with cost data from clinics to calculate the incremental costs of adding interventions. Using data from DCEs to make projections on the uptake of services to inform cost‐effectiveness analysis is valuable for intervention trials where resources do not exist to conduct the trial and obtain actual uptake data [29, 30, 31].

We determined the set of non‐dominated strategies for increasing the uptake of services and the order in which they should be implemented across the cost‐effectiveness frontier.

Friendliness, privacy and confidentiality are among the strongest drivers of choice for the use of HIV and contraceptive services among adolescents who participated in the DCE. We stratified the preferences identified from the DCE into modifiable and challenging to modify preferences. Friendliness and confidentiality were considered challenging to modify preferences―meaning that interventions to address these preferences are currently not well‐defined (and, therefore, not cost‐able). The DCE results and uptake projections show, however, that the challenging to modify factors are the most impactful and would provide the biggest increase in uptake―if an intervention to address these factors could be well‐defined and costed. Efforts aimed at addressing the attitudes of healthcare providers towards friendliness and confidentiality when delivering contraceptive and HIV services to adolescents can greatly enhance service utilization and, therefore, be a cost‐effective strategy [32, 33]. The impact is likely to go beyond just utilization of HIV and contraceptive services for adolescents into other health and educational outcomes [34, 35].

The effectiveness of value‐added services like access to free or affordable food, Wi‐Fi and cash transfers has been evaluated before [7, 33]. Although cost‐effective, it is important to be aware of their full impact and where this impact may plateau and new strategies must come in. Wi‐Fi and youth‐only services cost an incremental $7.01−$10.62 per additional adolescents expected to access the healthcare facility but on their own are only expected to increase adolescent utilization of health services by 9%. Should resources allow, additional considerations would be, first: Wi‐Fi and youth‐only services, second: the addition of subsidized food and third: the addition of afternoon clinic hours. Our results show that the order that we must implement interventions is the same between the two clinics and that altering the size of the clinic and other parameters may change the cost per additional adolescent utilizing services.

This study was subject to a number of limitations. Firstly, we were unable to cost the challenging to modify preferences―although these produced the largest impact. Studies that investigate the costs and effectiveness of interventions geared towards improving provider attitudes would be required to effectively evaluate the cost‐effectiveness of such interventions in this modelling framework. Behavioural economics‐informed interventions or nudges provide a potential way to influence provider attitude and behaviour [36]. Second, projections from the DCE are theoretical as they are based on stated preferences [37]. DCEs have, however, been used in other studies to project the uptake of services or products [30, 38]. Third, our study does not look at the heterogeneity of preferences and, therefore, differentiated impact and cost depending on the underlying population. Lastly, we assumed a linear scale‐up in costs with increasing reach of these services and we did not account for the saturation effect when interventions were used in combination. This may not reflect the stepwise increases in cost, but likely to reflect the likely cost‐effectiveness at scale.

The sensitivity analysis suggests that interventions that require more staff time or space, such as youth‐only or afternoon services, may not be as cost‐effective if uptake numbers are low. On the other hand, interventions like Wi‐Fi that benefit all participants, including additional adolescents, may be more cost‐effective.

## 5 CONCLUSIONS

Patient‐centred care has been expounded as the ultimate goal of healthcare service provision but is rarely a reality. The work described here might provide an approach to meet that need while considering resource limitations. Policy decision‐making requires consideration of both patient preferences and cost‐effectiveness information, and our study utilizes the DCE methodology to infer both. In instances where funding or time does not allow, the proposed methodology provides one mechanism to understand the possible cost‐effectiveness of different implementation scenarios. The results from this type of analysis can be useful in either guiding implementation―if no plan exists for further study―or could be used to guide study design for empirical cost‐effectiveness studies. Follow‐up work could look at the acceptability, feasibility and effectiveness of interventions built from these scenarios in different settings.

## COMPETING INTERESTS

The authors declare that they have no competing interests.

## AUTHORS’ CONTRIBUTIONS

CG and BEN conceptualized and designed the study. CGM conducted the analysis. CG prepared the original draft. CG, BEN, SP, LCL, CAR and AM reviewed and edited the draft.

## FUNDING

This study was made possible by the generous support of the American People and the President's Emergency Plan for AIDS Relief (PEPFAR) through the US Agency for International Development (USAID) under the terms of Cooperative Agreement 72067419CA00004 to Health Economics and Epidemiology Research Office. LL was supported by the National Institute of Mental Health of the National Institutes of Health under grant number K01MH119923.

## DISCLAIMER

The contents are the responsibility of the authors and do not necessarily reflect the views of the NIH, PEPFAR, USAID or the United States Government. The funders had no role in the study design, collection, analysis and interpretation of the data, in manuscript preparation or the decision to publish.

## Data Availability

The data that support the findings of this study are available from the corresponding author upon reasonable request.

## Sites

- South African non‐profit organization providing comprehensive PHC services provided by nurses and doctors. DOH support―accesses public‐sector drugs and laboratory tests.
- Government, PHC

Sites reported similar mean age, days in care and number of visits.

## Costing

- Resource use was calculated from the perspective of the provider, the public health system.
- All costs were updated to 2019 public‐sector prices and salaries.

**Table:**

Type of cost	Method for estimating cost
Variable costs (resources reported in subjects’ medical record)	
Diagnostics, supplies, other services reported in clinic register	Actual costs to the site, based on the most recent invoices.
Clinical staff time for service provision	Nurse, counsellor, at the study site and value at average total compensation rate for each level at study site.
Fixed costs (resources used for clinic operation, not allocated to individual subjects)	
Buildings, vehicles, equipment	Estimate total cost from market prices. Take proportion of depreciation plus maintenance and operating costs using standard step‐down costing approach. Divide by total number of HCT performed to estimate a cost per HCT.
Management and administration costs, including staff not providing direct patient care to individual subjects	Estimate salaries and other personnel and non‐personnel costs of non‐clinical staff (e.g. data clerks, cleaners, managers, etc.) and allocate per HCT as described above.
